# Repurposing Kir6/SUR2 Channel Activator Minoxidil to Arrests Growth of Gynecologic Cancers

**DOI:** 10.3389/fphar.2020.00577

**Published:** 2020-05-08

**Authors:** Daniela Fukushiro-Lopes, Alexandra D. Hegel, Angela Russo, Vitalyi Senyuk, Margaret Liotta, Gyda C. Beeson, Craig C. Beeson, Joanna Burdette, Ronald K. Potkul, Saverio Gentile

**Affiliations:** ^1^ Department of Pharmacology, Loyola University Chicago, Maywood, IL, United States; ^2^ Department of Medicine, University of Illinois Chicago, Chicago, IL, United States; ^3^ Department of Pharmaceutical Sciences, College of Pharmacy, University of Illinois Chicago, Chicago, IL, United States; ^4^ Department of Gynecologic Oncology, Loyola University Chicago, Maywood, IL, United States; ^5^ Department of Drug Discovery and Biomedical Sciences, Medical University of South Carolina, Charleston, SC, United States

**Keywords:** ion channels, cancer treatment, repurposing drug, minoxidil, oxidative stress, mitochondria

## Abstract

Gynecologic cancers are among the most lethal cancers found in women, and, advanced stage cancers are still a treatment challenge. Ion channels are known to contribute to cellular homeostasis in all cells and mounting evidence indicates that ion channels could be considered potential therapeutic targets against cancer. Nevertheless, the pharmacologic effect of targeting ion channels in cancer is still understudied. We found that the expression of Kir6.2/SUR2 potassium channel is a potential favorable prognostic factor in gynecologic cancers. Also, pharmacological stimulation of the Kir6.2/SUR2 channel activity with the selective activator molecule minoxidil arrests tumor growth in a xenograft model of ovarian cancer. Investigation on the mechanism linking the Kir6.2/SUR2 to tumor growth revealed that minoxidil alters the metabolic and oxidative state of cancer cells by producing mitochondrial disruption and extensive DNA damage. Consequently, application of minoxidil results in activation of a caspase-3 independent cell death pathway. Our data show that repurposing of FDA approved K^+^ channel activators may represent a novel, safe adjuvant therapeutic approach to traditional chemotherapy for the treatment of gynecologic cancers.

## Introduction

Gynecologic cancer is an uncontrolled growth and spread of malignant cells arising from the female reproductive tract. Although gynecologic cancers are a leading cause of death worldwide, they are under-studied. Endometrial cancer is the most common cancer of the female reproductive system for which the death rate has increased more than 100% in the past two decades. Whereas, ovarian/fallopian tube cancer, which mostly present the high-grade serous carcinoma (HGSC) histology type is the most lethal with a 5-year survival rate of only 30% and, more than 140,000 women die each year (Ries and Klastersky, 1986; Tomita et al., 2001; Miller et al., 2010; Zhang et al., 2014) worldwide because of disease progression. Also, ovarian cancer is a significant contributor to cancer disparities, since African-American women die at almost twice the rate as other racial groups (Mannhold, 2004).

Failure to treat these cancers is related to a variety of causes including late diagnosis and cancer heterogeneity. Also, the dose-limiting toxicity of available therapeutic agents can increase the morbidity and limits the ability to deliver the optimal therapeutic dose. Recently, targeted therapy has been evaluated to overcome resistant ovarian cancer but the lack of targets and the narrow therapeutic window results in an unfavorable risk/benefit profile. Ultimately, lack of population diversity on genomic database poses a serious limitation to translate research into practice (Landry et al., 2018). Consequently, it is important to identify novel targets and approaches to treat gynecologic cancers.

Potassium (K^+^) ion channels are integral membrane proteins that selectively allow an outflow of K^+^ ion to cross the cellular membrane. K^+^ channels have been traditionally studied for their role in controlling neuronal transmission, muscle contraction, or secretion. Nevertheless, recently studies have demonstrated that several members of the K^+^ channel family play a fundamental role in governing other critical cellular events such as proliferation (Wang, 2004; Beech and Cheong, 2006; Urrego et al., 2014; Bull and Doig, 2015; Rao et al., 2015; Gentile, 2016). Remarkably, cancers of different histotypes present alteration of specific K^+^ ion channel expression suggesting that these proteins could be critical factors in cancer biology.

**Figure 1:**
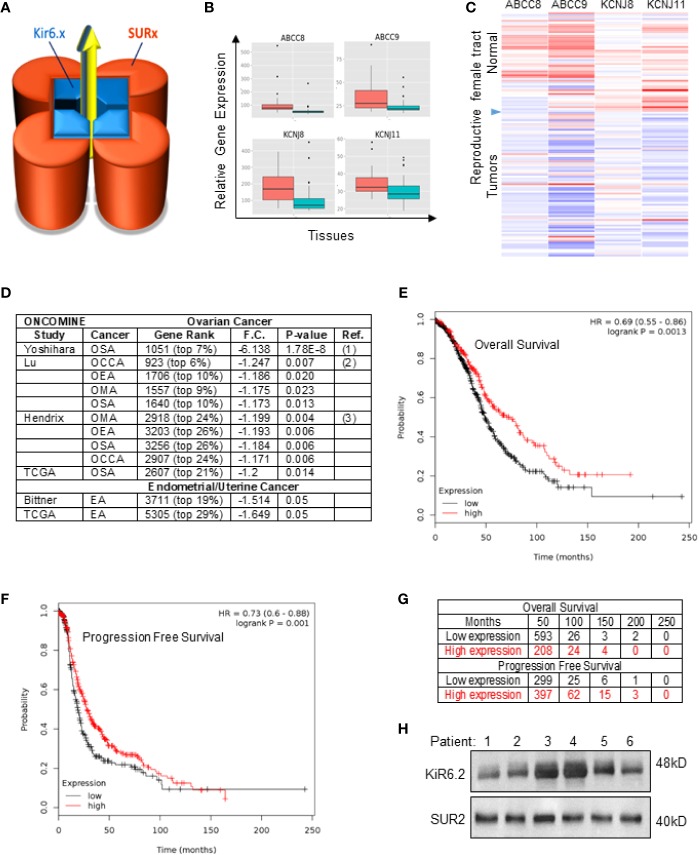
SUR2 expression and survival in patients with ovarian cancer. **(A)** Schematic representation of the Kir6/SUR potassium channel complex. **(B)** Boxplot and **(C)** Heat map representing analyses of copy number alteration for ABCC8, ABCC9, KCNJ8 and KCNJ11 genes in human primary tumors (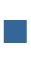) and normal tissues (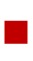) of the female reproductive system (merav.wi.mit.edu; criteria: p<0.5; fold change > 1.5). **(D)** Western blot showing expression of Kir6.2 and SUR2 proteins in 6 representative ovarian cancer tissues obtained from patients (Stage IV; IRB: LU209031, Loyola University Chicago) and **(E)** in SPEC-2, OVCAR-8 or OVCAR-4 human derived cell lines. **(F)** List of studies showing downregulation of the SUR2 gene in ovarian cancers compared to normal tissue (Oncomine.org dataset; threshold by: P-Value: 1–4; Fold Change: 2; Gene rank: Top 10%.). OSA, Ovarian Serous Adenocarcinoma; OCCA, Ovarian Clear Cell Adenocarcinoma; OEA, Ovarian Endometrioid Adenocarcinoma; OMA, Ovarian Mucinous Adenocarcinoma; Gene ranking, fold change (F.C.), p-value and probe used are reported. **(G)** Kaplan–Meier plots of overall survival and **(H)** progression-free survival in patients with ovarian cancer comparing the patients with high (red) and low (black) expression of SUR2 (top vs. bottom tertiles). For OS the median survival in low expression cohort = 49 months. Hazard ratios (HR) compare the hazard of relapse or death in the high expression versus low expression groups. Probe: Affy ID: 208462_s_at; Datasets: GSE14764, GSE23554, GSE26193, GSE26712, GSE30161, GSE3149, GSE32062, GSE63885, GSE9891, TCGA.

The Kir6/SUR potassium channel (**Figure 1A**) comprises a tetrameric structure in which four identical subunits Kir6.1 or Kir6.2 (encoded by the ABCC8 and ABCC9 genes respectively) are in complex with the sulfonylurea receptor SUR1 or SUR2 (encoded by the KCNJ8 and KCNJ11 genes respectively). While the Kir6 protein forms the channel pore, the SUR subunit is essential for controlling the opening and activity of the channel (Campbell et al., 2003; Mannhold, 2004). The presence of Kir6 subunit alone constitutes a non-functional channel.

Recent studies have revealed that the SUR subunit controls the selectivity of the pharmacological response to the drug that either inhibit or stimulate the Kir6/SUR channel. For example, glibenclamide (Glynase ®) acts as anti-diabetic medicine (type II diabetic patients) by inhibiting Kir6.1/SUR1 channel activity upon direct binding to the SUR1 subunit. Furthermore, minoxidil acts as an activator of the Kir6/SUR2 channel upon selective binding to SUR2 and does not generate significant side effects even at high doses. Minoxidil is approved by the FDA as anti-hypertensive agent (Loniten ®) and to arrest hair loss (Rogaine ®) (Bolduc and Shapiro, 2000; Rivera and Guerra-Tapia, 2008; Shorter et al., 2008). Remarkably, expression of different subunits of the Kir6/SUR complex presents tissue specificity. For example, neurons and pancreatic β-cells can express any of the Kir6 with SUR1 (but not SUR2) that contributes to electrical transmission or insulin secretion. In contrast, cardiac myocytes and vascular smooth muscle can express any of the Kir6 with SUR2 that contribute to protection against metabolic stress (Sato et al., 2004). Therefore, tissue specificity is a major contributor for the efficacy and safety (lack of significant side effects) of molecules that target SUR1 (e.g. Glynase) rather than SUR2 (e.g. Loniten).

Although drug discovery focusing on ion channels has generated an abundance of medicines of critical importance for treating human diseases, (Bull and Doig, 2015) the benefit of targeting the K^+^ channel in cancer has not yet been considered thoroughly. In our previous work, we have demonstrated that use of K^+^ channel activators can affect several hallmarks of cancer (Lansu and Gentile, 2013; Perez-Neut et al., 2015; Perez-Neut et al., 2016; Fukushiro-Lopes et al., 2018). In this study we found that gynecologic cancers express the Kir6.2/SUR2 K^+^ channel. We demonstrate that stimulation of the Kir6.2/SUR2 activity with the pharmacological activator Minoxidil (Loniten ®) produces cell death in endometrial and ovarian cancer cells which then translates into reduced tumor growth.

## Results

### Kir6.2/SUR2 Channel Is a Potential Favorable Prognostic Factor in Ovarian Cancer

In order to first determine the expression levels of potassium channel genes in gynecological cancers, a bioinformatic investigation was completed with the merav.wi.mit.edu (Shaul et al., 2016), Oncomine.org (Rhodes et al., 2004) which are curated gene expression database of publicly available microarray datasets. This study revealed that the KCNJ8, KCNJ11, ABCC8, ABCC9 genes are downregulated in cancer of the female reproductive tract when compared with the correspondent normal tissues (**Figures 1B, C**).

Interestingly, a differential expression analyses revealed that the ABCC9 (SUR2) gene is significantly downregulated in human ovarian cancer tissues independently of their histological characterization and in uterine cancer (**Figures 1C, D**). Analyses of the TCGA database showed that the SUR2 subunit appeared to be downregulated in at least 82% of the HGSC compared to healthy tissue (**Supplementary Figure 1B**). In contrast, KCNJ11 or KCNJ8 gene copy was unaltered.

To explore the clinical relevance of our findings, we conducted *in silico* analysis with the Kaplan-Meier Plotter database (KM plotter.com) (Nagy et al., 2018) by performing survival analyses based on selection of SUR2 as biomarker expression levels. This investigation revealed that high expression of the SUR2 gene is associated with improved overall survival (OS) in all ovarian cancer patients [Hazard Ratio (HR)= 0.7 (0.55-0.86); **Figures 1E–G**] with a 49% reduction in mortality and improved progression-free survival PFS [HR=0.73 (0.6–0.88); **Figure 1H**]. Subgroup analysis revealed that OS further improves in patients diagnosed with stage IV ovarian cancer [HR= 0.64 (0.5-0.84); **Supplemental Figure 1C**]. To further validate our bioinformatics study, we monitored protein expression level of the ion channels and functional subunits on ovarian cancer samples obtained from patient tissue donors that were diagnosed with stage IV high grade serous cancer. This investigation revealed that both Kir6.2 (KCNJ11) and SUR2 (ABCC9) proteins can be expressed in ovarian cancer (**Figure 1H**). Our data suggest that the KCNJ11 and SUR2 genes could be considered as a potential prognostic factor. Also, ovarian cancer patients could benefit from the pharmacological stimulation of the Kir6.2/SUR2 channel activity by minoxidil.

### Minoxidil Arrest Ovarian Cancer Tumor Growth

**Figure 2:**
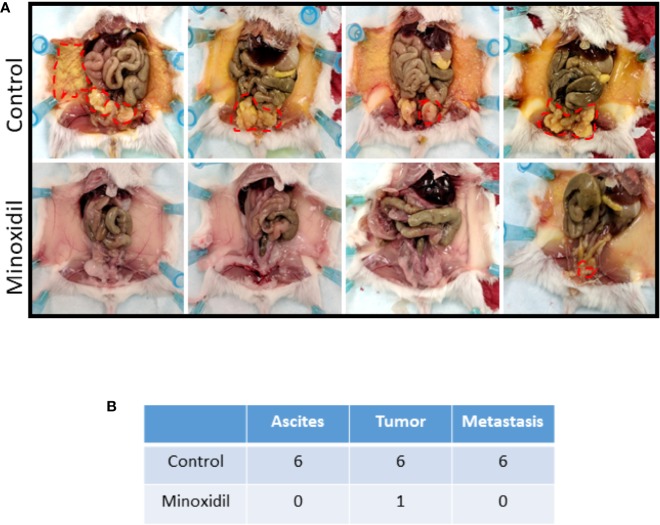
Effect of minoxidil on HGSC ovarian tumor development. **(A)** Representative mice with tumor burdens (red circle) treated with vehicle alone (Control) or with minoxidil (10 mg/kg). OVCAR8 cells were injected intraperitoneally into 12 female NSG mice (7 weeks old). Mice were separated randomly in two groups of six mice each. Two weeks after cell implant, one group of six mice was injected (IP; twice/wk) with 100 µl vehicle alone (DMSO) or minoxidil. White arrows indicate the presence of tumors. **(B)** Table listing the number of mice presenting ascites, tumor or metastasis (tumor formation in the peritoneum) in the control or treated groups.

To evaluate the relevance of our bioinformatics investigation, we established a xenograft model of Kir6.2/SUR2 positive HGSC cell line (**Supplementary Figure 1**) in the NOD-SCID-IL2Rγnull (NSG) (Villanueva et al., 2013) mice in which we assessed the effect of minoxidil (**Figure 2**). As expected, 6 weeks after cell implant, all control mice (untreated) presented ascites fluid (mean volume of 2.8 ml). Dissection of the control mice revealed that all mice presented a primary tumor and hundreds of tumor nodules that had developed on the peritoneal wall. In contrast, in five of the six mice that were treated with minoxidil, no measurable amount of ascites, tumor or metastasis were produced (10mg/Kg; **Figures 2A, B**). Mice treated with higher dose of minoxidil (50 mg/Kg) were severely stressed after four injections as they presented limited motility, inappetence, loss of weight and lethargic behavior and were sacrificed before the end of the study. The total weight of the only primary tumor found in the minoxidil treated group was estimated to be decreased of 90-fold when compared with the tumor of the untreated mouse. These experiments reveal that the Kir6.2/SUR channel activator minoxidil inhibited ovarian tumor development.

### Minoxidil Does Not Alter Cardiac Function in NSG Mouse Harboring Ovarian Cancer

**Figure 3:**
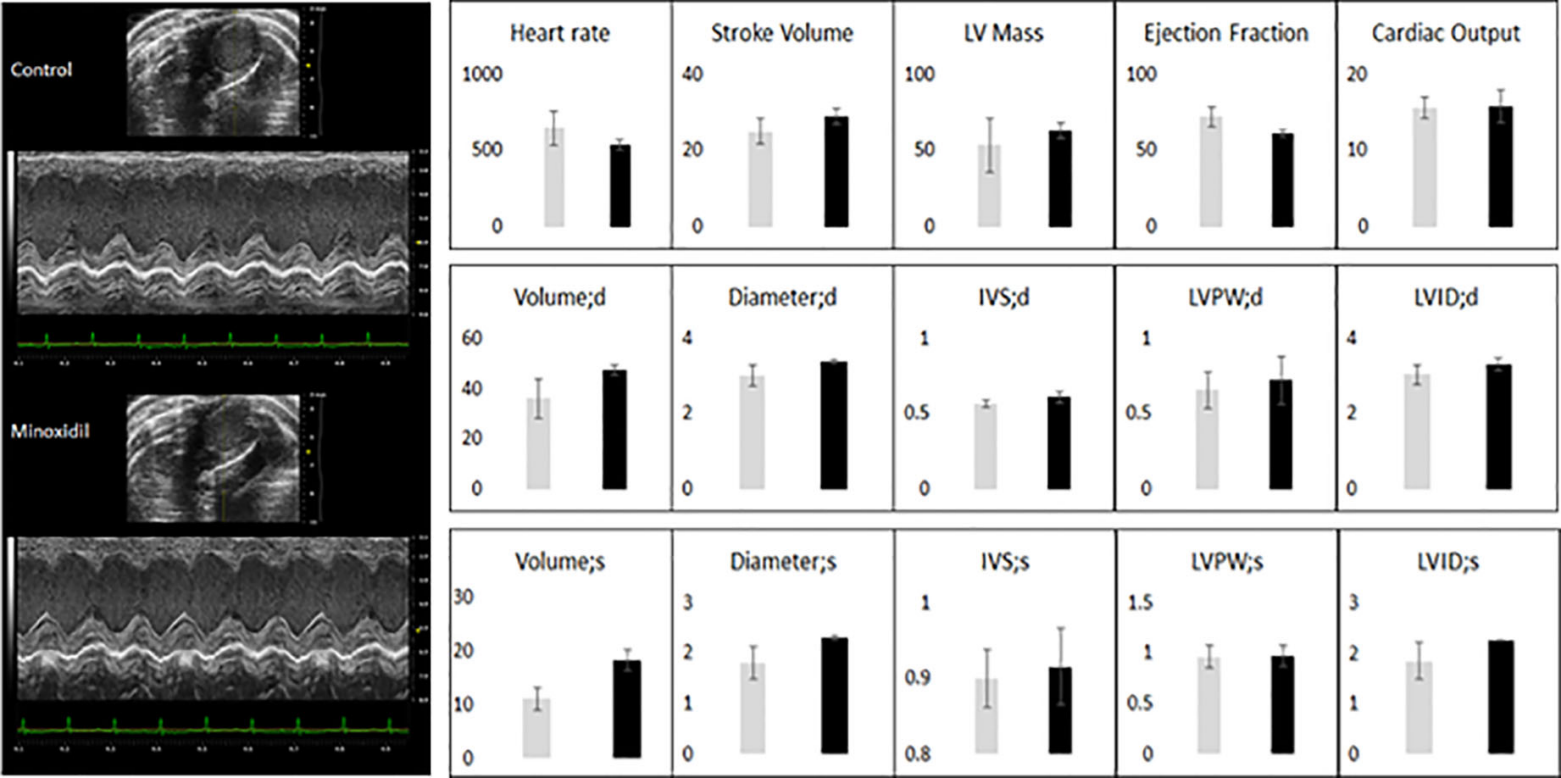
Effect of minoxidil on *in vivo* cardiac function. Transthoracic M-mode echocardiograms echocardiography (Vevo 2100 with MS400 series transducer, Visualsonics Inc.) of (A) control (n = 3) and minoxidil-treated mice (n = 3). Figure represents a motion mode of the left ventricle, which is obtained with a single ultrasound beam transmitted through the heart with the resulting image displayed over time. Light Gray bar = control; Dark gray bar = minoxidil. HR, heart rate (beats per min); EF, ejection fraction (%); LVIDs & LVIDd, left ventricular internal diameter systole and diastole; LVAWs & LVAWd, left ventricular posterior wall systole and diastole (mm). Beat-to-beat detection was achieved using preset detection and analysis settings for mice (typical QRS width 10 ms, R waves > 60 ms apart, Pre-P baseline 10 ms, Maximum PR 50 ms, Maximum RT 40 ms, ST height at 10 ms, averaging four beats). Averaged ECG measurements were taken from a minimum of 20 beats per mouse. ECG analysis was performed in LabChart8 (ADInstruments).

Cardiotoxicity related to drugs that are used during anticancer treatments can often limit the benefit of anticancer therapy and can cause interruption of the treatment. Minoxidil is considered a safe drug as it has been widely used both topically (Rogaine ®) and systemically (Loniten ®) in humans worldwide without generating significant side effects (Pettinger, 2017). Nevertheless, minoxidil has never been tested as a therapeutic agent against ovarian cancer and the potential cardiotoxicity of minoxidil in cancer patients is unknown. To address this potential issue, we used transthoracic echocardiograms (TTA) to evaluate cardiac performance in mice baring ovarian cancer (**Figure 3**). TTA was performed within 2 h or 25 days after exposure to minoxidil to measure respectively acute and chronic response to the drug.

Notably, we found that minoxidil did not produce a change in any of the parameters measured suggesting that minoxidil can be considered a safe antiproliferative agents against ovarian cancer.

### Minoxidil Inhibits Proliferation and Arrests the Cell Cycle

**Figure 4:**
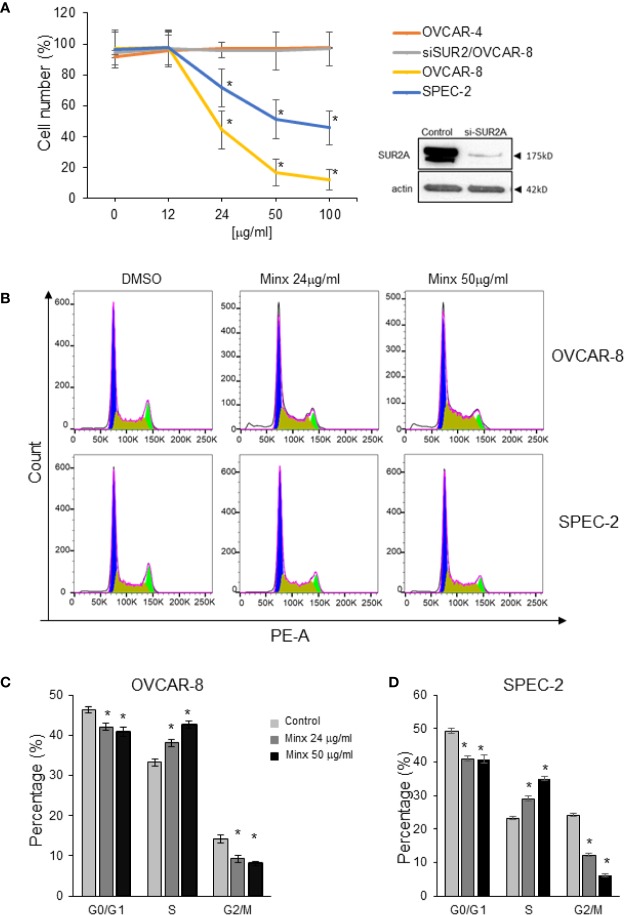
Effect of minoxidil on cell proliferation. **(A)** Percentage of viable cells (MTS assay, Promega) of OVCAR-4, OVCAR-8, OVACR8 with siRNA targeting SUR2 (siSUR2/OVCAR-8), and SPEC-2 exposed to different concentration of minoxidil for 24 h. (To compare data points from different treatment groups, ANOVA and post hoc Dunnett’s tests were used. n=6; * P<0.005) and Western blot showing the efficacy of si-RNA in reducing SUR2 expression. **(B)** Representative results showing minoxidil-dependent accumulation in the S phase of the cell cycle of ovarian (OVCAR-8) and uterine (SPEC-2) cancer cell lines. Sub-confluent cells were treated with different concentration of minoxidil or DMSO 24 h and then flow cytometry was performed to analyze cell cycle. **(C, D)** Quantification of the effect of minoxidil on cell cycle Minoxidil. Data represent mean±SEM (n=3; * p<0.001; unpaired t-test).

To understand the mechanism that underlies the inhibitory effect of minoxidil on ovarian tumor growth, we monitored the effects of minoxidil on proliferation of Kir6.2/SUR2 positive or Kir6.2/SUR2 negative cell lines (**Figure 4A** and **Supplementary Figure 1**).

We found that application of minoxidil (24 h) strongly inhibited proliferation rate in OVCAR-8 and SPEC-2 cell lines in a concentration-dependent manner (**Figure 4A**). In contrast, proliferation OVCAR-4 which do not express SUR2 was not affected by minoxidil at any concentration (**Figure 4A**). To gain insights on the specificity of minoxidil we produced an OVCAR-8 cell line in which expression of SUR2 was suppressed by si-RNA (siSUR2-OVCAR-8). As expected, minoxidil did not significantly alter the proliferation rate of these cells indicating that the inhibitory effect of minoxidil is mediated specifically by SUR2.

To better characterize the mechanism through which minoxidil suppresses ovarian tumor growth, we monitored changes in the cell cycle with flow cytometry (**Figure 4B**). Kir6.2/SUR2 positive or Kir6.2/SUR2 negative cell lines were treated with minoxidil or DMSO (control) for 24 h. Next, DNA content in cells from each plate was analyzed with flow cytometry by propidium iodide staining. We found that in both Kir6.2/SUR2 positive cell lines (OVCAR-8 and SPEC2) minoxidil produced a depletion of cells in G0/G1 and in G2/M phase and, an accumulation of cells in S phase of the cell cycle in a dose-dependent manner (**Figures 4B–D**). In contrast, in the Kir6.2/SUR2 negative cell line OVCAR-4, minoxidil did not produce any significant changes in the distribution of cells in different cell cycle phases (**Supplementary Figure 1D**)

It has been established that the entry of cells into mitosis is regulated by important factors such as CDK1 and WEE1 kinases and by cyclin B. Remarkably, we found that minoxidil inhibited CDK1 activity as indicated by the increased phosphorylation of Y15 on CDK1 (**Supplementary Figure 1E**), inhibited WEE1 activity as indicated by reduced phosphorylation of S642 on WEE1 and produced the accumulation of cyclin B.

Our experiments indicate that pharmacological stimulation of the Kir6.2/SUR2 potassium chanel activity with minoxidil inhibited cell proliferation by arresting the cell cycle in the G2/M phase.

### Minoxidil Alters Oxidative State by Affecting Mitochondria

**Figure 5:**
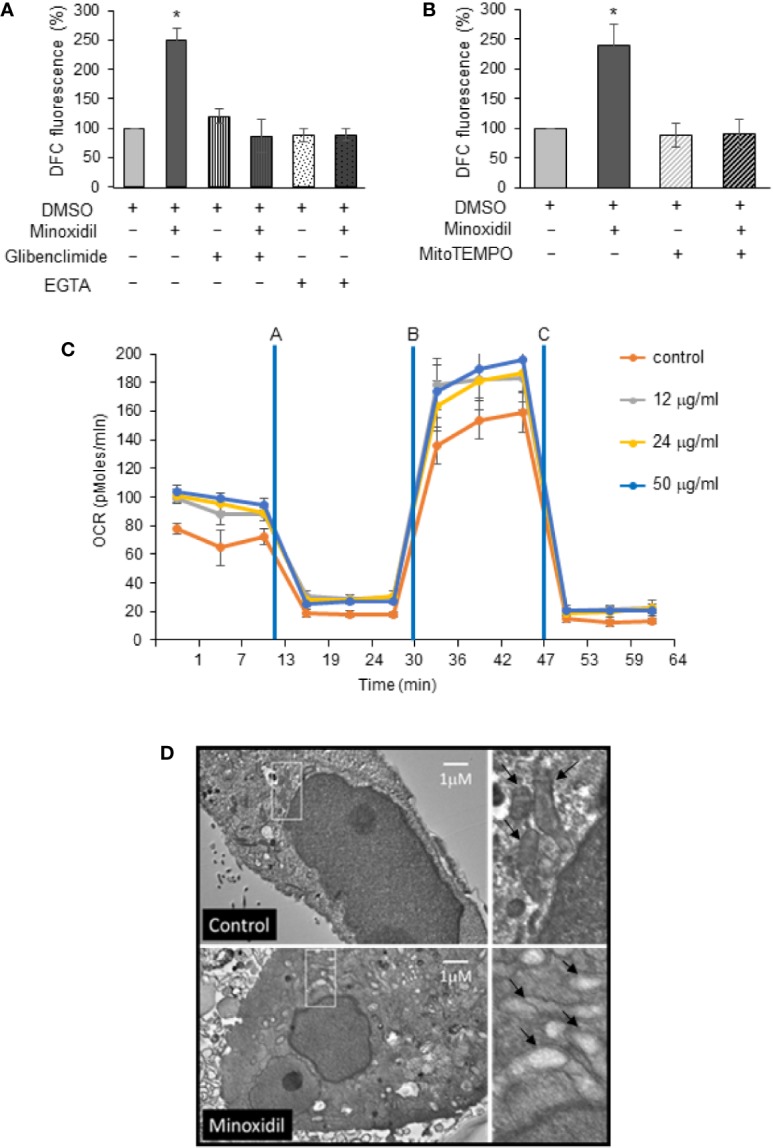
Minoxidil disrupts mitochondria function. **(A)** Effect of minoxidil alone (50 µg/ml) or with the Kir6.2/SUR blocker glibenclimide (10 µg/ml) or the Ca^2+^ ion chelator EGTA (2.4 µg/ml) or **(B)** with MitoTEMPO (5 µg/ml L) compared to control (DMSO) on cellular ROS formation in human-derived OVCAR-8 (DCFH-DA to 2′,7′-dichlorofluorescein DCF, Thermo Fisher Sci; Fluorescence was analyzed in a plate reader (PHERAstar FS, BMG LABTECH) with excitation at 485 nm and emission at 520 nm). Data is expressed as mean ± SEM; *p < 0.001. **(C)** Kinetic Oxygen Consumption Rate (OCR) of OVCAR-8 cells to minoxidil at 0, 12, 24, or 50 µg/ml. Cells were plated at 25,000/well in XF24 V7 culture plates. The assay medium was the substrate-free base medium. n=3; (Assay design, data analysis, and file management were performed with Agilent Seahorse Wave Desktop software) **(D)** Representative electron microscopy micrographs showing mitochondria in OVCAR-8 cells treated with minoxidil (50 µg/ml; 24 h). The disruptive effect of the drug is shown as lack of cristae in the mitochondria of treated cells (white box enlarged in right panel).

We have previously demonstrated that stimulation of a K^+^ channel activity in cancer cells can alter the cellular oxidative state in a Ca^2+^ entry-dependent manner. Hence, to gain insight into the Kir6.2/SUR2-dependent mechanism of ovarian cancer growth arrest, we monitored the reactive oxygen species (ROS) level in cells treated with minoxidil. We found that minoxidil significantly increased ROS concentration (**Figure 5A**) when compared to untreated cells. In contrast, application of the Kir6 channels blocker Glibenclamide completely abated the effect of minoxidil on ROS production (**Figure 5A**) confirming that the effects of minoxidil is mediated by Kir6 currents. Also, application of the cell impermeable Ca^2+^ chelator, EGTA to the growth medium suppressed the effect of minoxidil on ROS production (**Figure 5A**) indicating that minoxidil-dependent ROS production is mediated by extracellular Ca^2+^.

ROS production are generally associated with stressed mitochondria and it underlies oxidative damage that can lead to cell death. Remarkably, we found that use of the specific mitochondria superoxide scavenger, MitoTEMPO completely inhibited the minoxidil-dependent ROS production (**Figure 5B**) and cell death (**Supplementary Figure 2A**). These data suggest that minoxidil alters the cellular oxidative state by affecting mitochondria. To further test this hypothesis, we performed a Mito Stress Test assay with a Seahorse Analyzer with cells treated with or without minoxidil.

Interestigngly, we found that minoxidil alters mitochondrial function (**Figure 5C**) in a concentrantion-dependent manner. Much of the ATP utilization in cells is committed to maintenance of the Na^+^/K^+^ gradient via the plasma membrane Na/K-ATPase. The intracellular depletion of K^+^ that is caused by activation of Kir6.2/SUR2 via minoxidil is then restored by activation of Na/K-ATPase. This event creates an increased ATP demand while lowering the intracellular K^+^ ion concentration and thereby enhancing Ca^2+^ load on the mitochondria.

Collectively these effects should increase basal ATP production via mitochondria as seen in the minoxidil promoted increase in basal oxygen consumption rate (OCR) (**Figure 5C**). Increased flux through the electron transport chain will also increase leak of electrons to oxygen resulting in enhanced formation of superoxide ions. The enhanced leak due to minoxidil exposure is most evident when the cells are uncoupled with the carbonyl cyanide-p-trifluoromethox-phenyl-hydrazon (FCCP), minoxidil-treated cells have a higher OCR, and the higher leak when comparing the oligomycin—Antimycin/Rotenone rates that directly measure leak. Collectively, these results suggest that efflux of K^+^ due to minoxidil increases ATP demand, leading to higher leak and, thus, higher superoxide levels as suggested by the ROS dye and TEMPO data.

Also, we caried out an electron microscopy analyses to monitor the effect of minoxidil on mitochondria integrity. We found that cancer cells exposed to minoxidil for 24 h presented a severe mitochondrial morphological abnormality (**Figure 5D**) when compared to untreated cells suggesting that the inhibitory effects of minoxdil on ovarian cancer growth is mediated by alteration of mitchocondria integrity.

**Figure 6:**
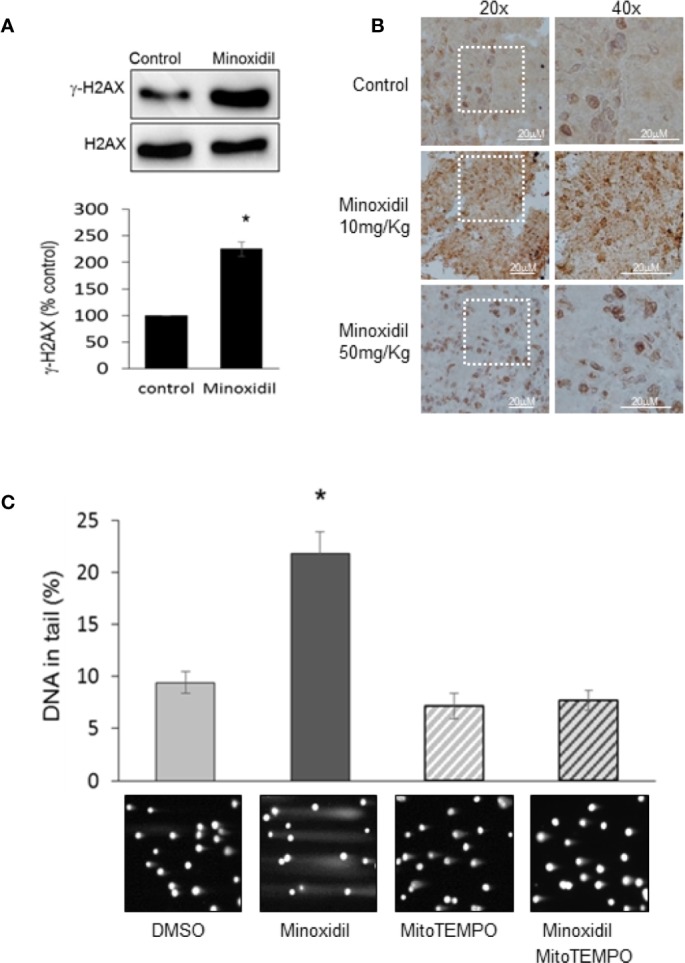
Minoxidil produces DNA damage. **(A)** Western blot analyses of the DNA damage sensor γH2AX in OVCAR-8 cells treated with minoxidil (50 µg/ml) versus control or **(B)** in tumor extracted from mice treated with minoxidil vs control. **(C)** Bar chart representing the effect of minoxidil alone or in combination with MitoTEMPO on DNA integrity. Data is expressed as mean ± SEM; *p < 0.05. Bottom panels are representative images of cells subjected to neutral comet assay are presented below the bars corresponding to specific treatments.

A major consequence of increased ROS production is alteration of DNA integrity. Therefore, we investigated the effects of minoxidil on DNA damage. Our Western blot analyses of OVCAR-8 cells line (**Figure 6A**) and immunohistochemistry examination of the tumors extracted from mice (**Figure 6B**) revealed that minoxidil significantly increased DNA damage as indicated by increased protein level of the DNA damage marker γH2AX. These data were confirmed by a comet assay which indicated that minoxidil produced a significant increse of double breaks DNA damage. In contrast, in cells treated with the Mitochondrial ROS buffer, MITOTEMPO, the effect of minoxidil on DNA damage (**Figure 6C**) was completely suppressed.

These data suggest that pharmacological stimulation of the Kir6.2/SUR2 complex severely affects mitochondria and DNA integrity in ovarian cancer cells.

### Minoxidil Produces Caspase-3 Independent Cell Death

**Figure 7:**
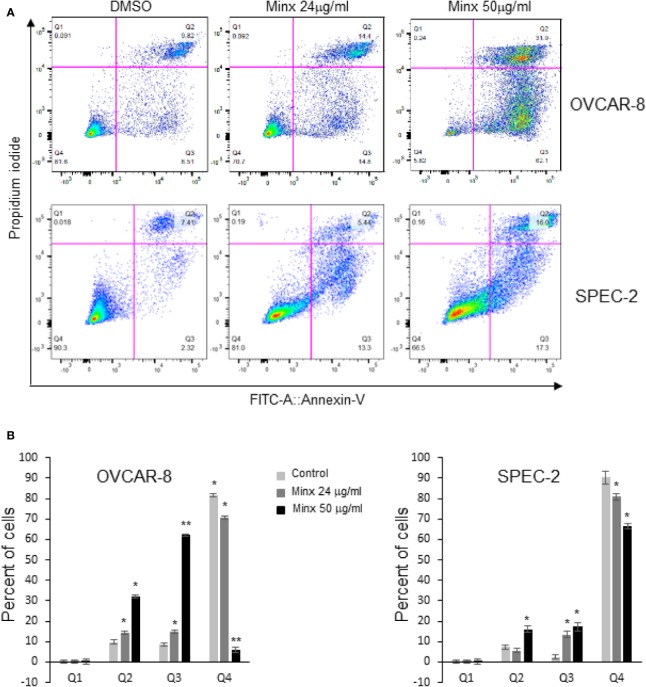
Minoxidil activates cell death. **(A)** Apoptotic analysis of OVCAR-8 or SPEC-2 cells by flow cytometry. Cells were treated with different concentrations of minoxidil alone for 24hr and incubated with AV-FITC and PI. Stained cells were analyzed by Flow cytometry. Percentage of intact cells (AV-/PI−) and different stages apoptotic cells (AV+/PI−, AV+/PI+ and AV-/PI+) are presented. **(B)** Quantification of the apoptotic effect of minoxidil. Data represent mean±SEM (n=3; *p<0.001; ** p<0.0001; unpaired t-test).

**Figure 8:**
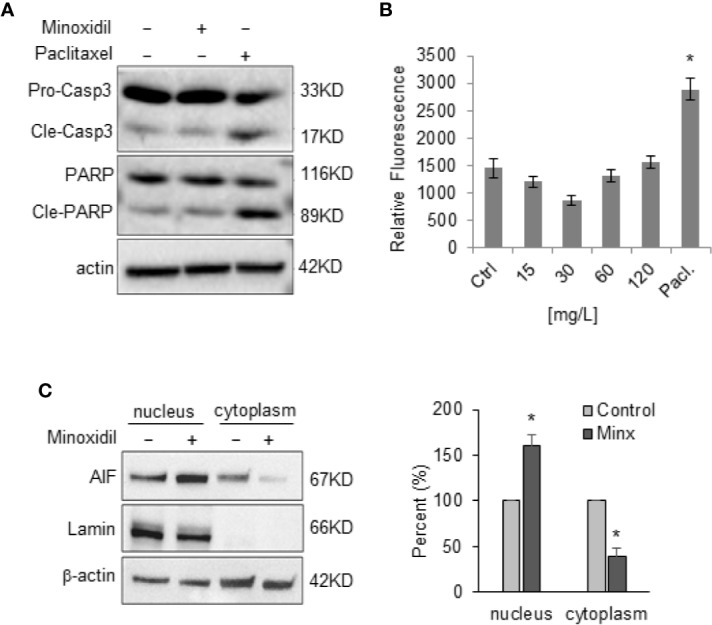
Minoxidil fails to activate casase-3. **(A)** Representative Western blots of caspase-3 and PARP proteins in OVCAR-8 cells treated with minoxidil for 24 h (n=3). The procaspase-3, PARP and their cleaved products are indicated. **(B)** ELISA assay showing the effect of different concentration of minoxidil on caspase activity. Paclitaxel was used as control for caspase activity. To compare data points from different treatment groups, ANOVA and post hoc Dunnett’s tests were used. n=3 plates; *p<0.005. **(C)** Western blot analyses of AIF protein level in nuclei and cytoplasm of OVCAR-8 cells treated with minoxidil (50 µg/ml/24 h) and quantification of percent change compared to control *p<0.01.

To understand whether the effects of minoxidil associated with cell death we performed a bi-parametric cytofluorimetric assay by using a fluorescein isothiocyanate (FITC)-conjugated annexin V (AV) and propidium iodide (PI) protocol. Our experiments revealed that minoxidil induced the increase of the percentage of early (Q3 = AV+/PI-) and late apoptotic cells (Q2=AV+/PI+; **Figures 7A, B**). In contrast, treatment with mitoTEMPO completely abolished the effect of minoxidil on cell death (**Supplementary Figure 2**). However, minoxidil treatment failed to produce activation of the apoptotic executioner caspase-3 as indicated by the lack of cleaved caspase-3 and cleaved PARP and, by the caspase-3 activity ELISA assay (**Figures 8A, B**). As control for caspase-3 activity, a separate group of cells were treated with paclitaxel which produced the expected activation of caspase-3. These results show that minoxidil produces cell death in a caspase-independent manner.

Caspase-3 independent cell death can occurr via accumulation of the apoptosis-inducing factor (AIF) into the nucleus which leads to DNA fragmentation. Thus, we monitored the effect of minoxidil on AIF. Our Western blot analyses of AIF expression levels in cell fractions revealed that minoxidil determined a significant increase of AIF protein level in the nucleus (**Figures 8B, C**).

In conclusion, our data demonstrate that the Kir6.2/SUR2 potassium channel could be considered as a potential prognostic factor of cancers cells originated from the female reproductive tract. Also, pharmacological stimulation of the Kir6.2/SUR2 potassium channel activity with the FDA approved molecule minoxidil arrests ovarian tumor growth by arresting the cells in the G2/M phase of the cell cycle. This event associates with alteration of the cellular oxidative state, disruption of the mitochodndria and DNA structure and activatio of a caspase-3 independent cell death.

## Discussion

The current study found that selective K^+^ channels activators can severely affect viability of ovarian cancer and multiple hallmarks of cancer (Lansu and Gentile, 2013; Perez-Neut et al., 2015; Rao et al., 2015; Fukushiro-Lopes et al., 2018). This suggests that pharmacologically targeting K^+^ channels could be considered as a potential anticancer strategy.

Importantly, low expression of the SUR2 gene correlates with poor overall survival in ovarian cancer patients. These data suggest that the SUR2 gene could be a valuable prognostic factor for the most lethal of all gynecologic cancers. Furthermore, ovarian cancer patients could benefit from drugs that stimulate the Kir6.2/SUR2 channel activity and the expressio of the channel may help to stratify patinets for response to the drug. When systemically applied, minoxidil (Loniten ®) is quickly metabolized in minoxidil sulfate, which acts as a selective activator of the Kir6.2 channel upon binding with its constitutive partner SUR2 in the arteries (Mfuh and Larionov, 2015) without producing neurological side effects or insulin disorders due to the absence of the drug target in the brain or pancreas (Mfuh and Larionov, 2015). Experiments demonstrated that application of 10 mg/Kg of minoxidil arrested ovarian tumor growth in NSG mice. In a separate group of NSG mice harboring ovarian tumor, injection of 50 mg/Kg resulted to be toxic.

The dose of minoxidil has been decided based on previously reported experiments demonstrating that 50 mg/Kg body weight (BW) is the maximally tolerated dose in a healthy mouse (Khanna et al., 2011) and on pharmacokinetic studies in humans (Fleishaker and Phillips, 1989). Due to patient-to-patient variation in blood levels, it has been difficult to establish an overdosage level of Loniten ®. At the best of our knowledge, the highest non-lethal blood concentration of minoxidil that has been reported is of ≈3.5 µg/ml (≈3.3 mg/Kg) which correlates to approximately double the maximum suggested dose for anti-hypertensive therapy (100 mg/day; ≈1.6 mg/Kg) (Kikuchi et al., 2016). A recent well-established method to translate the dose of drugs used from one animal species to another has been published (Reagan-Shaw et al., 2008) (http://dtp.nci.nih.gov). On the basis of this formula, the human equivalent dose of 10 mg/Kg of minoxidil in the mouse is 0.8 mg/Kg in a person (average of 60 Kg) which is ≈75% lower than the maximum amount of drug suggested dose of 100 mg/d recommended in the Physician’s Desk Reference.

Expression of the Kir6.2/SUR2 channel in the mammalian cardiac myocytes might raise the concern that minoxidil can cause alteration of the heart function. Interestingly, in our experiments we did not observe any significant change of cardiac performance in mice baring ovarian tumors and treated with 10 mg/Kg minoxidil acutely or chronically. Also, previous studies have shown that minoxidil preserves cardiac myocytes by stress damages rather than impinge on cardiac function.

Our in vitro investigation to understand the mechanism underlying the inhibitory effect of minoxidil on ovarian cancer growth revealed that minoxidil significantly altered the production of superoxide species (ROS) in cells. ROS are chemically reactive molecules derived by the reduction of oxigen. Several cellular resources of ROS have been identified but, as mitochondria are actively involved in the production of energy using oxygen, this organelle produce the largest amount of ROS. Ultimately, ROS production is finely controlled by a variety of mechanisms that include changes in intracellular Ca^2+^ concentration and antioxidant activity. Minoxidil significantly increased the oxigen consuption rate (OCR), which was abated by using a ROS scavenger that localizes in the mitochondria. We have previously demonstrated that stimulation of K^+^ channel activity in non-excitable cells leads to Ca^2+^ entry which in turn can affect mitochondria-dependent ROS production (Fukushiro-Lopes et al., 2018). As expected, the combination of minoxidil with a Ca^2+^ chelator, which limits the available extracellular Ca^2+^ to enter the cells, significantly suppressed ROS formation when compared with minoxidili alone. These data show that minoxidil-dependent ROS production is mediated by the mitochondria.

Oxidative cellular stress can occur when the amount of ROS overwhelm the antioxidant mechanisms, which can result in damaging several cellular compartments. Stimulation of the Kir6.2/SUR2 channel with minoxidil produces a catastrophic event that include mitochondria disruption and severe DNA damage. These events associate with activation of a cell-death pathway that is not mediated by the canonical apoptodic executioner caspase-3 or by necrosis as indicated by our annexin V assay. Nonetheless, to better understand that death pathway activated by minoxidil further experiments need to be performed. Also, in addition to the localization at the surface membrane, the minoxidil sensitive Kir/SUR complex has been found expressed in several cell compartments including th sarcoplasmic reticulum (Shorter et al., 2008), endoplasmic reticulum (Salari et al., 2015), mitochondria (Garlid and Halestrap, 2012), and endosome/lysosomes (Bao et al., 2011). Therefore, at this time, we cannot state that the anticancer effect of minoxidil is exclusively mediated by the surface membrane Kir6/SUR2 complex other studies are necessary to confirm our data.

## Materials and Methods

### Tumor Xenograft Models

Two million OVCAR-8 ovarian cancer cells were intraperitoneally (IP) injected in NOD-scid-IL2Rγnull (NSG) female mice (n=12) of 5–6 weeks old. Mice were locally bred and were a generous gift from Michael Nishimura. Mice were randomly divided into two groups (n=6/group) and treated with vehicle (DMSO) or minoxidil via i.p. twice per week. Tumor development was measured every 5 days and mice were sacrificed 6 weeks after the initial minoxidil treatment. This study was reviewed and approved by Loyola University Chicago Institutional Animal Care and Use Committee guidelines (Animal Assurance No. D16-00074). Minoxidil was purchased from Tocris Bioscience [Cat. No. 0583; purity ≥99% (HPLC)].

### Effect of Gene Expression Levels on Patient Survival

We performed a meta-analysis to explore the association of *SUR2* gene expression with survival in ovarian cancer patients using KM plotter (KMplotter.org). This web-based application uses a manually curated database that combines all publicly available microarray gene expression data sets. Meta-analyses are performed by integrating gene expression data with clinical data. Patients are segregated into low vs. high expression cohorts, and Kaplan-Meier survival plots with log-rank p values are generated, along with hazard ratios (HR) and 95% confidence intervals (CI). For the meta-analysis of *SUR2* expression, we interrogated the KM plotter database for Affymetrix probe ID: 208462_s_at, and the expression data was partitioned into tertiles. Analyses compared the top and bottom tertile groups.

### Cell Culture, Antibodies, and Reagents

OVCAR-4, OVCAR-8 human ovarian cancer cell lines were obtained from the American Type Culture Collection (ATCC; Manassas, VA). SPEC-2 cells were a generous gift of Dr. Ram Ganapathi (University of North Carolina Charlotte) and maintained 231 cells (ATCC) was maintained in Dulbecco’s modified Eagle’s medium (DMEM) (4.5 g/L glucose) supplemented with 10% fetal bovine serum (FBS), Penicillin (100 ug/ml)/streptomycin (100 ug/ml) antibiotics. Cells were incubated under humidified conditions with 5% CO_2_ at 37°C. All antibodies for Western blot analyses were purchased from Cell Signaling Technologies, Inc (Boston, MA).

### Respirometry Assay

OVCAR-8 cells were plated into XF96^e^ plates at a 50% confluence for 24 h. After the first 24 h the cells were treated with minoxidil (Loniten ®; Rogaine ®) at three different concentrations (0, 60, 120, 240 µM) for the next 24 h. The media was exchanged to a phosphate buffered the assay MEMS modified to contain no bicarbonate and 1 mM phosphate to better measure the acidification rates of the cells. After the cells reached 85% confluence, they were assayed on the XF^e^96 instrument (Agilent technologies) using a 3 min measure—2 min mix cycle. The oxygen consumption rates (OCR) and extracellular acidification rates were measured with the Mitochondrial Stress Assay, using 1 µM Oligomycin, 1 µM FCCP, and 100 nM Rotenone/100 nM 2 µM Antimycin A. Each treatment was for 15 min during which the OCR and ECAR rates were measured.

### Western Blot Analysis, Immunoprecipitation, and Immunofluorescence Staining

Cells were harvested and lysed with cold radioimmunoprecipitation assay (RIPA) buffer [50 mM Tris HCl (pH 8.0), 150 mM NaCl, 1% NP-40, 0.5% sodium deoxycholate, 0.1% SDS, 1 mM phenylmethylsulfonyl fluoride (PMSF), 1 mM sodium fluoride (NaF), 1 mM sodium orthovanadate (Na3VO4), and protease inhibitor cocktail]. An equal amount of protein samples were subjected to SDS-polyacrylamide gel electrophoresis (PAGE) and transferred onto a nitrocellulose membrane. Membranes were blocked with 5% nonfat milk in Tris-Buffered Saline (TBS) containing 0.1% Tween 20 (TBST), incubated with primary and secondary antibodies, and detected using Super Signal West Pico Chemiluminescent Substrate (Thermo Scientific, Pittsburgh, PA). Immunoprecipitation was performed by incubating protein lysates with primary antibodies at 4°C overnight, followed by incubation with protein A/G-conjugated agarose beads (Santa Cruz Biotechnology, Santa Cruz, CA). Beads were washed with cold RIPA buffer, resuspended in 2x SDS sample buffer, boiled for 5 min, and subjected to SDS-PAGE, followed by western blot analysis. Cells were grown on poly D-Lysine coated coverslips and treated with or without minoxidil. Following treatment, cells were washed with 1x PBS, fixed with acetone:methanol fixation, blocked with 5% bovine serum albumin (BSA) for 1 h at room temperature, and incubated with primary antibodies, followed by incubation with Alexa Fluor 594- or Alexa Fluor 488-conjugated secondary antibody (Life Technologies, Carlsbad, CA). Next, slides were incubated with 50 ng/ml DAPI to stain the nuclei. The coverslips were mounted on VECTASHIELD reagent (Vector Laboratories, Burlingame, CA) and fluorescent images were taken using confocal microscopy (Carl Zeiss Meditec, Inc., Thornwood, NY). Cells incubated with 1% BSA alone were served as negative controls.

### Nuclear/Membrane Fractionation

Cells were washed, lysed with a hypotonic buffer [10 mmol/L 4-(2-hydroxyethyl)-1-piperazineethanesulfonic acid (HEPES) buffer (pH 7.9), 1.5 mmol/L MgCl2, 10 mmol/L KCl, 10% glycerol, 0.5 mmol/L PMSF, and 1 mmol/L dithiothreitol (DTT)], and incubated on ice for 15 min. Five μl of 10% NP-40 was added to each lysate. After centrifugation at 13,200 rpm for 10 min, the supernatant was collected as the membrane/cytosolic fraction. The nuclear pellet were lysed with buffer solution containing 20 mmol/L HEPES buffer (pH 7.9), 1.5 mmol/L MgCl2, 400 mmol/L NaCl, 0.2 mmol/L EDTA, 20% glycerol, 0.5 mmol/L PMSF, and 1 mmol/L DTT, and collected by centrifugation at 13,200 rpm for 10 min at 4°C.

### Detection of Reactive Oxygen Species (ROS)

Cells were seeded at 4 × 104 cells/ml in 96 well plates. Adhered cells were incubated in the dark with DCFH-DA to 2′,7′-dichlorofluorescein (1 h/37°C; 40 μM, ThermoFisher Sci). Cells were washed and subjected to different drug treatments for 2h. Fluorescence was analyzed in a plate reader (PHERAstar FS, BMG LABTECH) with excitation at 485 nm and emission at 520 nm.

### DNA Damage Detection

Tumors from mice that received DMSO or minoxidil were excised and processed for cryosectioning. 12 mm thick sections were stained with DAPI (100 ng/μl) and imaged using a Leica SPE confocal microscope (Leica Microsystems). The number of nuclei exhibiting fragmented versus normal morphology was counted manually in ImageJ software. Data are expressed as a fraction of the normalized total number of nuclei.

Neutral comet assay was performed for detecting DNA double-strand breaks. The images were captured on a fluorescent microscope and quantified by using TriTek CometScore™.

### Echocardiogram and ECG Analysis

Cardiac function was assessed using echocardiography (Vevo 2100 with MS400 series transducer, Visualsonics Inc.). Briefly, mice were anesthetized with 1.2%–1.5% isoflurane and placed on a heating pad to maintain body temperature. Parasternal short-axis 2-D echocardiograms and M-mode cine loops were taken at the level of the papillary muscles. The total length of anesthesia was less than 10 min. Evaluation of stored data was performed offline by a sonographer blind to treatment groups. ECG analysis was performed in LabChart8 (ADInstruments)

### Immunohistochemistry (IHC)

Tumors isolated from control mice (DMSO) and one mouse treated with minoxidil were fixed with PFA, embedded in paraffin and sectioned with microtome. Antigene retrieval from tissue sections was performed using sodium citrate, blocked in appropriate serum and incubated overnight with γH2AX (Cell Signaling Technology) at 37C and then with secondary biotynilated antibodies for 30 min at RT. Couterstaining with hematoxylin was performed. Tissues were observed with a Zeiss Axioscope 2 and digital images were acquired with Zeiss Axiocam digital camera and Axiovision software.

## Conclusions

Our work adds important insights on the effects of pharmacological activation of K^+^ channel for the treatment of cancer. Due to the considerable limited genomic/proteomic data, animal model for different gynecologic cancers and the extended heterogeneous nature of cancers of the female reproductive system we cannot predict at this time what percent of the cancer patients would benefit from using minoxidil. However, in consideration of the limited therapeutic options for ovarian cancers, we propose that repurposing a well-known, readily available and low-cost non-cancer drug as minoxidil might provide a possible rapid transition from bench to clinic and offer an opportunity to address existing unmet patient needs.

## Author’s Note

This work is dedicated Estella (Lidia) Cronk, Nicoletta De Canale and Craig Beeson. 'Anything in existence, having somehow come about, is continually interpreted anew, requisitioned anew, transformed and redirected to a new purpose.' (F. Nietzsche).

## Data Availability Statement

The datasets generated during and/or analyzed during the current study are available from the corresponding author on reasonable request.

## Ethics Statement

The studies involving human participants were reviewed and approved by IRB: LU209031, Loyola University Chicago. The patients/participants provided their written informed consent to participate in this study. The animal study was reviewed and approved by IRB: LU209031, Loyola University Chicago Institutional Animal Care and Use Committee guidelines (Animal Assurance No. D16-00074).

## Author Contributions

DF-L, AH, VS, GB, and CB have contributed in the design and execution of the experiments in the reported study. AR, VS, ML, JB, RP, and SG have contributed in the conception and interpretation of the data in the reported study. ML, JB, and SG have contributed in writing the manuscript.

## Funding

This research was funded by The Cronk Foundation.

## Conflict of Interest

The authors declare that the research was conducted in the absence of any commercial or financial relationships that could be construed as a potential conflict of interest.

The funders had no role in the design of the study; in the collection, analyses, or interpretation of data; in the writing of the manuscript, or in the decision to publish the results.

## Supplementary Material

Supplementary Figure 1
**(A)** PCR analyses of Kir6.1, Kir6.2, SUR1, SUR2A and SUR2B in the ovarian cancer cells OVCAR-4, OVCAR-8 and SPEC-2. **(B)** Percent of tumor samples from the TCGA database with under/over expression of SUR2 gene. Different cut-offs of expression z-scores are indicated. **(C)** Kaplan–Meier plots of overall survival in patients with ovarian cancer comparing the patients with high (red) and low (black) expression of SUR2 (top vs. bottom tertiles). Probe: Affy ID: 208462_s_at; Datasets: GSE14764, GSE23554, GSE26193, GSE26712, GSE30161, GSE3149, GSE32062, GSE63885, GSE9891, TCGA. **(D)** Representative results showing the effect of minoxidil in the SUR2 negative cell line OVCAR-4. **(E)** Western blot analyses of proliferation markers phosphorylated Wee1, phosphorylated CDK1 and cyclin A in OVCAR-8 treated with or without minoxidil (50mg/ml; 24hr).

Supplementary Figure 2
**(A**, **B)** FACS (PI) analysis showing the rescue effect of MitoTEMPO on the lethal effect of minoxidil on OVCAR-8 cells.
