# Supplementary figures and images for: Repurposing Kir6/SUR2 Channel Activator Minoxidil to Arrests Growth of Gynecologic Cancers
Source: Front Pharmacol. 2020 May 8;11:577. doi: 10.3389/fphar.2020.00577 (PMC7227431; doi:10.3389/fphar.2020.00577)

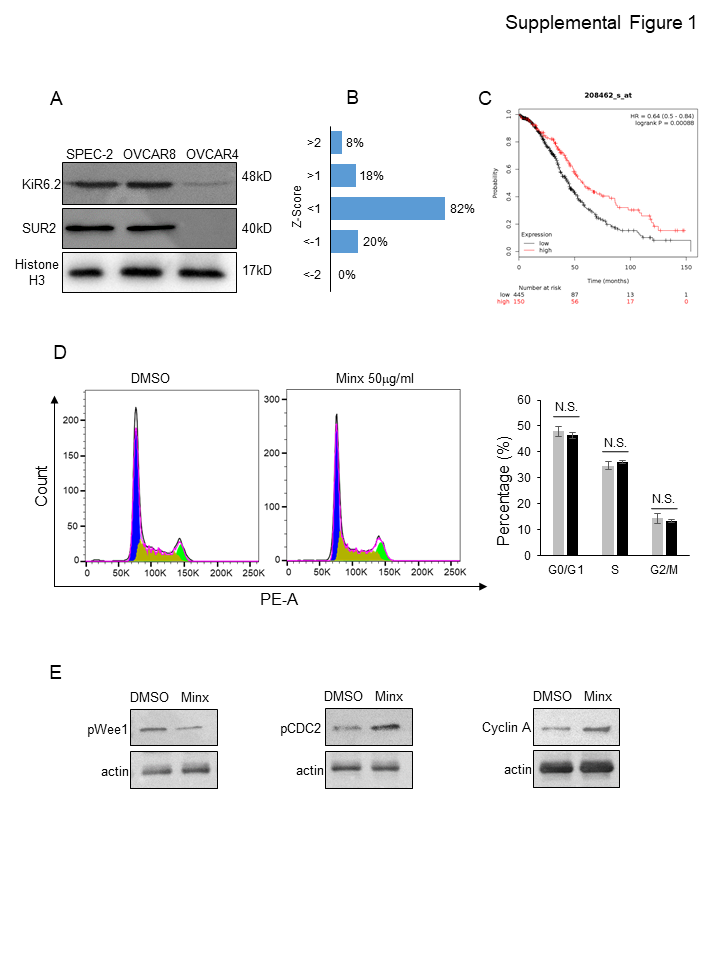

Supplement: Supplementary Figure 1 — (A) PCR analyses of Kir6.1, Kir6.2, SUR1, SUR2A and SUR2B in the ovarian cancer cells OVCAR-4, OVCAR-8 and SPEC-2. (B) Percent of tumor samples from the TCGA database with under/over expression of SUR2 gene. Different cut-offs of expression z-scores are indicated. (C) Kaplan–Meier plots of overall survival in patients with ovarian cancer comparing the patients with high (red) and low (black) expression of SUR2 (top vs. bottom tertiles). Probe: Affy ID: 208462_s_at; Datasets: GSE14764, GSE23554, GSE26193, GSE26712, GSE30161, GSE3149, GSE32062, GSE63885, GSE9891, TCGA. (D) Representative results showing the effect of minoxidil in the SUR2 negative cell line OVCAR-4. (E) Western blot analyses of proliferation markers phosphorylated Wee1, phosphorylated CDK1 and cyclin A in OVCAR-8 treated with or without minoxidil (50mg/ml; 24hr). [file Image_1.tif]

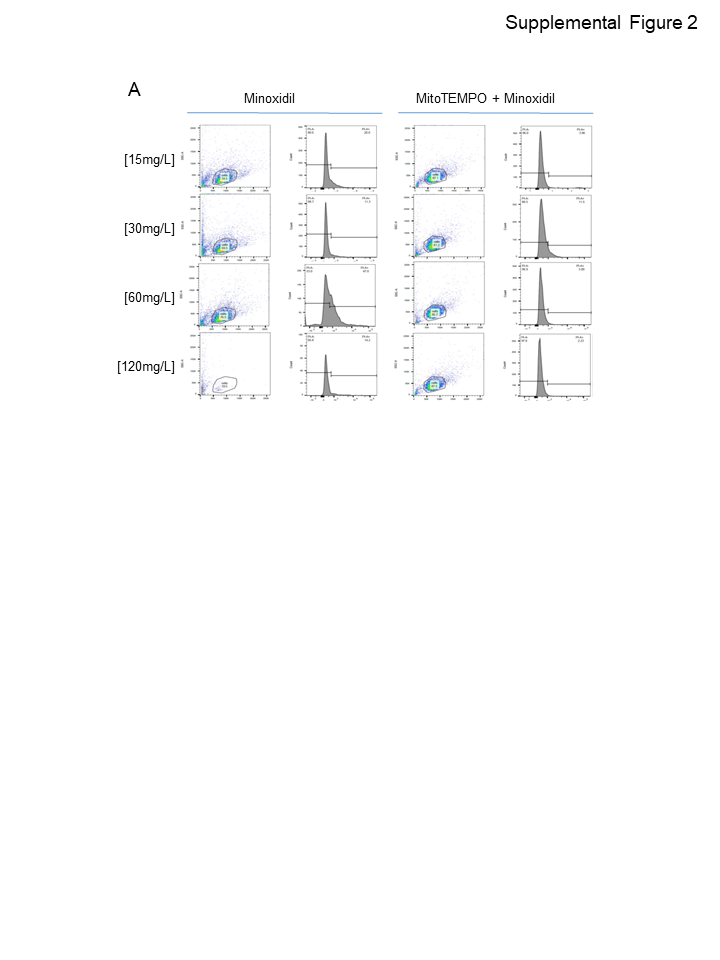

Supplement: Supplementary Figure 2 — (A, B) FACS (PI) analysis showing the rescue effect of MitoTEMPO on the lethal effect of minoxidil on OVCAR-8 cells. [file Image_2.tif]
